# “Can I afford to live today?” The emotional toll of navigating the healthcare system with type 1 diabetes

**DOI:** 10.3389/fendo.2025.1555265

**Published:** 2025-03-24

**Authors:** Shiraz Harel, James Lukasik, Annabelle Wilcox, Kasia J. Lipska, Stuart A. Weinzimer, Sakinah C. Suttiratana, Laura M. Nally

**Affiliations:** ^1^ Department of Pediatrics, Yale School of Medicine, New Haven, CT, United States; ^2^ Department of Psychiatry, Stanford University, Stanford, CA, United States; ^3^ Bryn Mawr College, Bryn Mawr, PA, United States; ^4^ Hartwick College, Oneonta, NY, United States; ^5^ Department of Pediatrics, University of Utah, Salt Lake City, UT, United States; ^6^ Physician Associate Program, Yale School of Medicine, New Haven, CT, United States; ^7^ Section of Endocrinology, Department of Internal Medicine, Yale School of Medicine, New Haven, CT, United States; ^8^ Office of Health Equity Research, Yale School of Medicine, New Haven, CT, United States

**Keywords:** type 1 diabetes, emotions, social media, barriers, healthcare access, diabetes distress, diabetes supplies, financial stress

## Abstract

**Objectives:**

People with diabetes (PWD) face significant barriers to accessing insulin and diabetes supplies, including high prices, leading some to turn to social media for material support. This study explores emotions among PWD who have relied on assistance from social media networks when navigating access to diabetes medications and supplies (DMS).

**Methods:**

We conducted a mixed methods study of individuals with type 1 diabetes (T1D) and their caregivers who have used social media to obtain DMS. Participants were recruited through social media based on self-reported use of social media to obtain diabetes support. Transcripts of semi-structured, telephone interviews were analyzed and categorized, and consensus discussions resolved discrepancies and refined definitions of themes.

**Results:**

Thirty individuals (mean age 31+/- 8 years, 29 female, 5 caregivers) were interviewed. The analysis revealed four categories of emotions: anxiety and stress, fear of health problems and financial consequences, frustration with the healthcare system, and feelings of powerlessness and vulnerability. Nearly all interviewees reported anxiety or stress due to the financial burden of managing diabetes and fear for their or their child’s health and safety. Diabetic ketoacidosis, unnecessary bodily harm or sickness, or fear of dying due to running out of insulin worried participants. Most participants described the process of obtaining DMS to be more stressful than their daily diabetes management.

**Conclusions:**

PWD described strong negative emotions related to navigating the healthcare system and acquiring DMS. Policy changes are urgently needed to support to individuals with type 1 diabetes in order to enhance their quality of life, guarantee equitable access to care, and cultivate a more compassionate and inclusive healthcare system. The reported magnitude of stress is notable, especially given the attention typically focused on the stress of diabetes management.

## Introduction

The first use of insulin in 1922 changed diabetes from a death sentence to a manageable chronic illness, but decades of rising insulin prices have changed the narrative. Just over 5% of adults in the United States live with type 1 diabetes (T1D) and depend on insulin to survive (1). In recent years, high out of pocket costs have made diabetes medication and supplies inaccessible to many (2, 3). As a result, as many as 1 in 4 patients have been forced to ration insulin, and many are forced to find alternative methods of obtaining their diabetes medications and supplies (DMS) (4). Many patients have turned to alternative sources, including social media and other medical suppliers outside of the healthcare system, to acquire necessary medication and supplies (5–7).

Published reports have described PWD engaging in an underground exchange of medications and supplies out of necessity, as well as crowdsourcing to raise money to afford these life-sustaining tools (5, 7, 8). These non-traditional pathways include trading medications or supplies on online diabetes community forums, making requests on social media platforms such as X (formerly Twitter), Facebook, or Instagram, buying and selling in the underground market, or reaching out to organizations that help people find access to diabetes medication or supplies. However, there is limited research about the individual experiences of the people relying on social media networks and the consequential emotional toll. In this study, we describe the emotional experiences of people with type 1 diabetes who have used social media to acquire their DMS.

## Materials and methods

### Study design

We conducted a cross-sectional mixed-methods study of PWD and their caregivers who have reached out to social media at any time to obtain DMS. Our objective was to describe the emotional experiences involved with obtaining and affording DMS. Participants were recruited from June 30, 2021 to March 10, 2022 through social media (Facebook, Instagram, Twitter) and advocacy organizations (Mutual Aid Diabetes, The Embrace Foundation, T1International). Telephone interviews were conducted with individuals with T1D at least 18 years of age living in the United States and parents or caregivers of youth with T1D who had reached out through social media for DMS. We chose this population because we wanted to describe the scenarios that led people with T1D to use non-traditional methods like social media to obtain their necessary DMS. Individuals were excluded if they could not read, understand, or speak English.

The study was approved by our institutional Investigational Review Board, and a waiver of signed consent was obtained. Participants completed an eligibility screen over the telephone and provided electronic consent or verbal telephone consent prior to study participation. We created the interview guide by defining our objectives and developing open-ended questions that were understandable at an 8^th^ grade reading level. The interview guide was reviewed by members of the research team, clinicians who see patients with T1D, and adults in the community who live with T1D. Participants completed a semi-structured interview and verbally responded to 16 demographic questions and 4 additional questions (**Supplementary Data Sheet 1**). Demographic questions addressed age, sex, race, ethnicity, income, A1c, as well as financial and educational information. Participants were also queried about stress and financial issues related to acquiring diabetes supplies and its impact on their health using a 5-point Likert scale (1 = “No stress”, 5 = “Severe stress”). Participants were asked about specific situations when they turned to social media to acquire DMS, the obstacles that they encountered that led them to social media, and the emotions they experienced. Interviews were conducted over the telephone, audio recorded, transcribed verbatim, and all identifying information removed. Each participant received an electronic gift card for $20 for participation.

The interview and coding team reflected on their personal experiences and biases, acknowledging their positions of privilege and the fact that some team members live with T1D, which may influence conducting interviews and interpretating the data. The research team was multidisciplinary with a broad range of expertise, including pediatric and adult endocrinology, public health, as well as social and cultural factors that impact health.

We used inductive qualitative research methods, allowing themes and patterns to emerge directly from the interviews. Themes from the interviews were independently coded by 4 members of the research team (S.H., A.W., J.L., and L.M.N.) using NVivo (Lumivero, Denver, CO), and collaborative thematic analysis was performed, where each code was discussed and agreed upon by 2 team members. Differences in coding among researchers were discussed and resolved through a consensus model approach. Thematic data analyses proceeded after data collection. Emerging themes and patterns were examined within and across the interviews with respect to feelings about the process of obtaining DMS. Answers to quantitative questions are reported using descriptive statistics.

## Results

**Figure 1:**
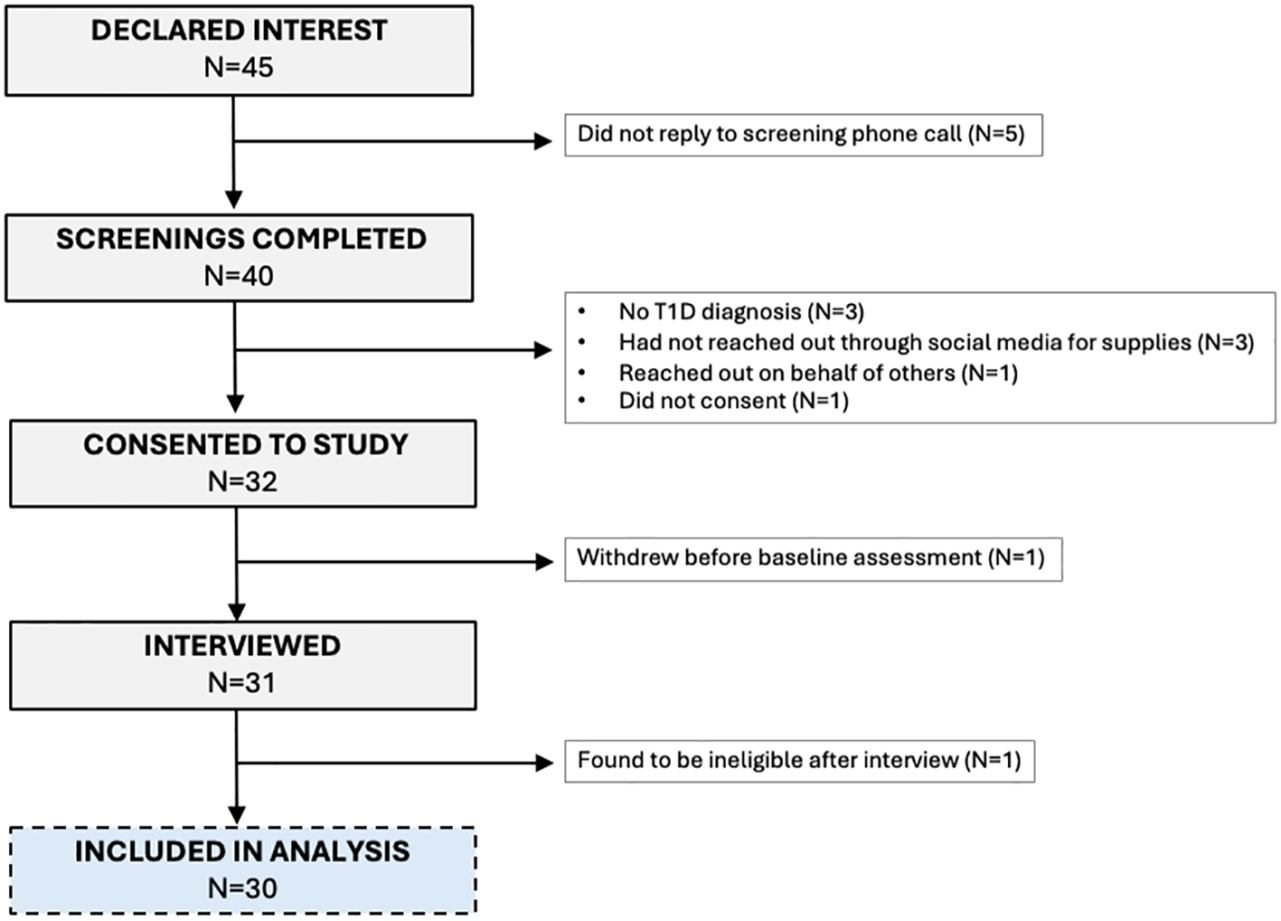
Consort diagram depicting participant selection and inclusion in the analysis.

**Figure 2:**
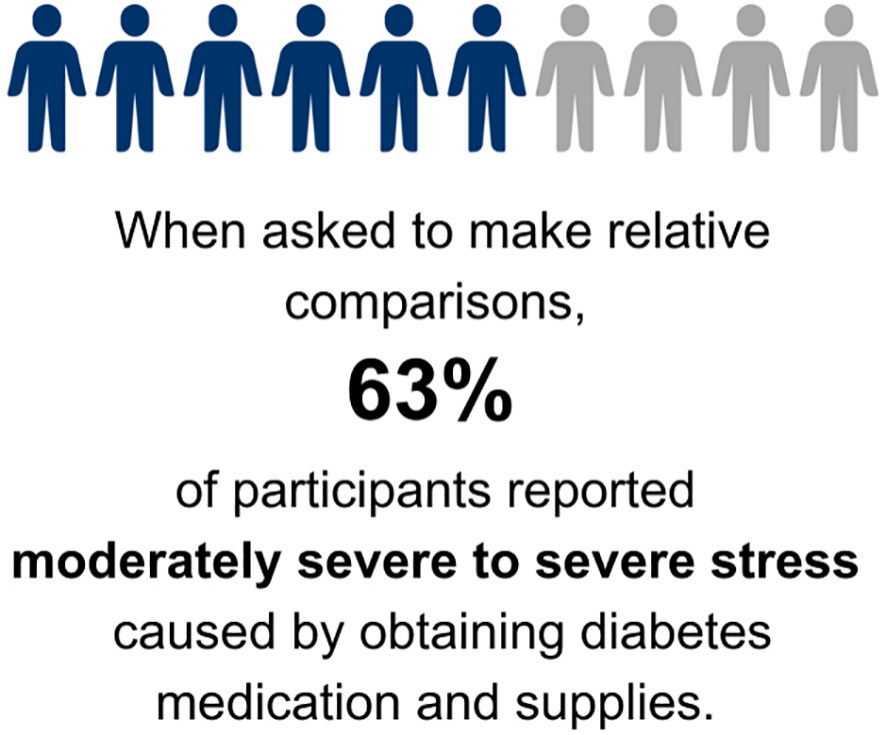
Definitions of salient themes based on emotional responses that individuals with diabetes or caregivers of youth with diabetes experienced. Icons were purchased individually and modified under a commercial license.

**Figure 3:**
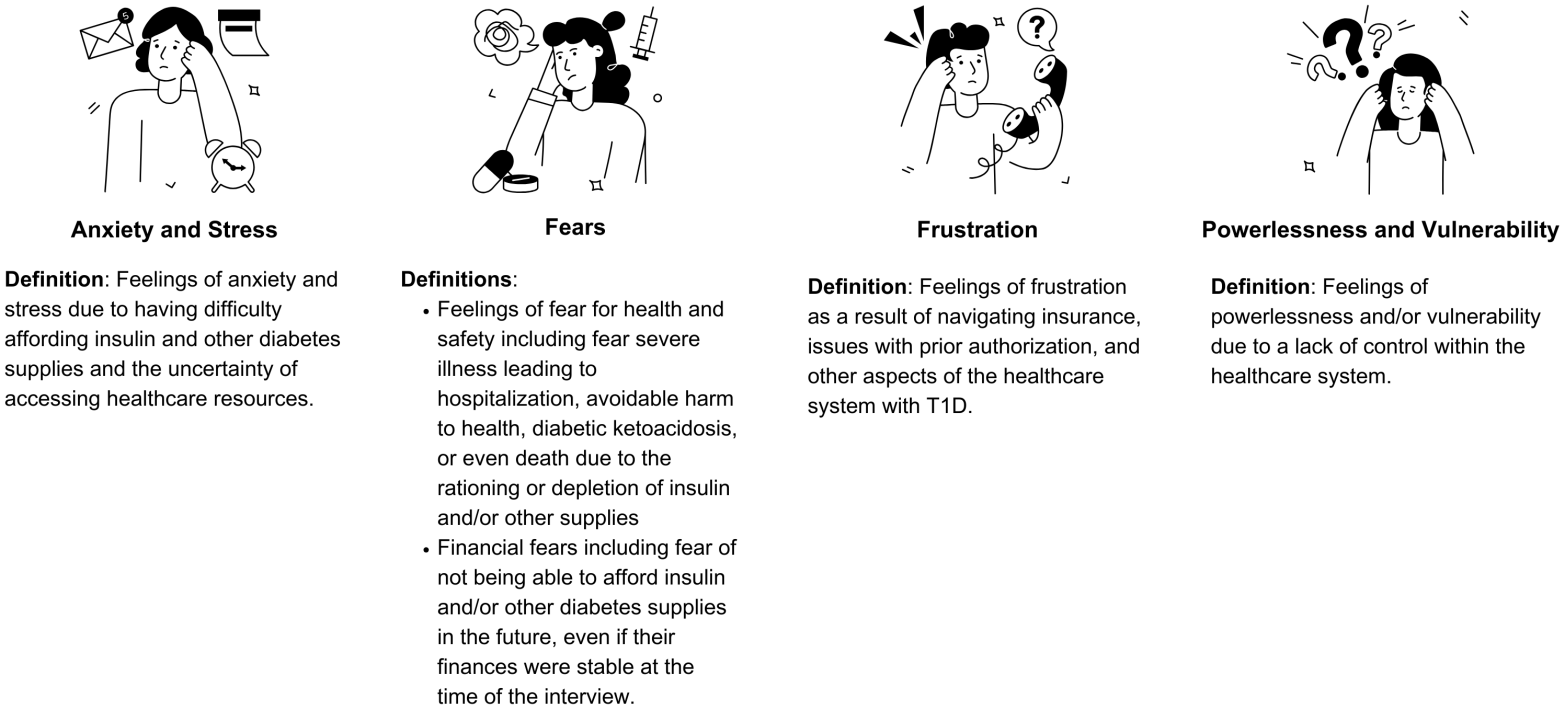
Most prominent themes and subthemes related to reaching out to barriers encountered when trying to access diabetes-specific healthcare.

Of the 45 participants who expressed interest in the study, 31 were interviewed and 1 was found to be ineligible after the interview (**Figure 1**). Participants were primarily non-Hispanic white (63%), female (97%); 63% had private health insurance, 17% had public health insurance and 13% had a combination of private and public health insurance at the time of the interview (**Tables 1**, **2**). Emotions are one of five broad content areas used to categorize participants’ narrative responses. Within this content area, four salient themes emerged: (1) anxiety and stress; (2) fear for health problems or health consequences; (3) frustration that results from navigating insurance issues and the healthcare system; and (4) feelings of powerlessness and vulnerability (**Figures 2**, **3**).

**Table 1:** Demographic characteristics of those interviewed.

Characteristic	Details
Caregivers of youth with T1D	5 (17%)
Age (years)	31 ± 8 (22-52)
Sex (female)	29 (96%)
Born in United States	29 (97%)*
Highest level of education	
High school diploma	1 (3%)
Some college but no degree	7 (23%)
Associate’s degree	2 (7%)
Bachelor’s degree	12 (40%)
Master’s degree	7 (24%)
Professional degree	1 (3%)
Household Income	
<$25,000	8 (26%)
$25,001 - $50,000	8 (26%)
$50,001 - $100,000	9 (30%)
>$100,000	5 (17%)

Categorical variables are listed as n, %. Continuous variables are listed as mean ± SD (range).

*1 person reported being born in Germany.

**Table 2:** Demographic characteristics of those with diabetes.

Characteristic	Details
Adults	25 (83%)
Children	5 (17%)
**Age (years)**	27 ± 8 (6-44)
Sex (female)	26 (87%)
Duration of T1D (years)	15 ± 10 (1-42)
HbA1c (%)	7.2 +/- 1.5 (4.7 - 10.3)
Race	
Caucasian	22 (73%)
African American and American Indian or Alaska Native	1 (3%)
American Indian or Alaska Native	1 (3%)
No race reported	6 (20%)
Ethnicity	
Hispanic or Latinx	9 (30%)
Non-Hispanic or Latinx	21 (70%)
Health Insurance	
Public and Private	4 (13%)
Private	19 (63%)
Public	5 (17%)
None	2 (7%)

Categorical variables are listed as n, %. Continuous variables are listed as mean ± SD (range).

**Figure 4:**
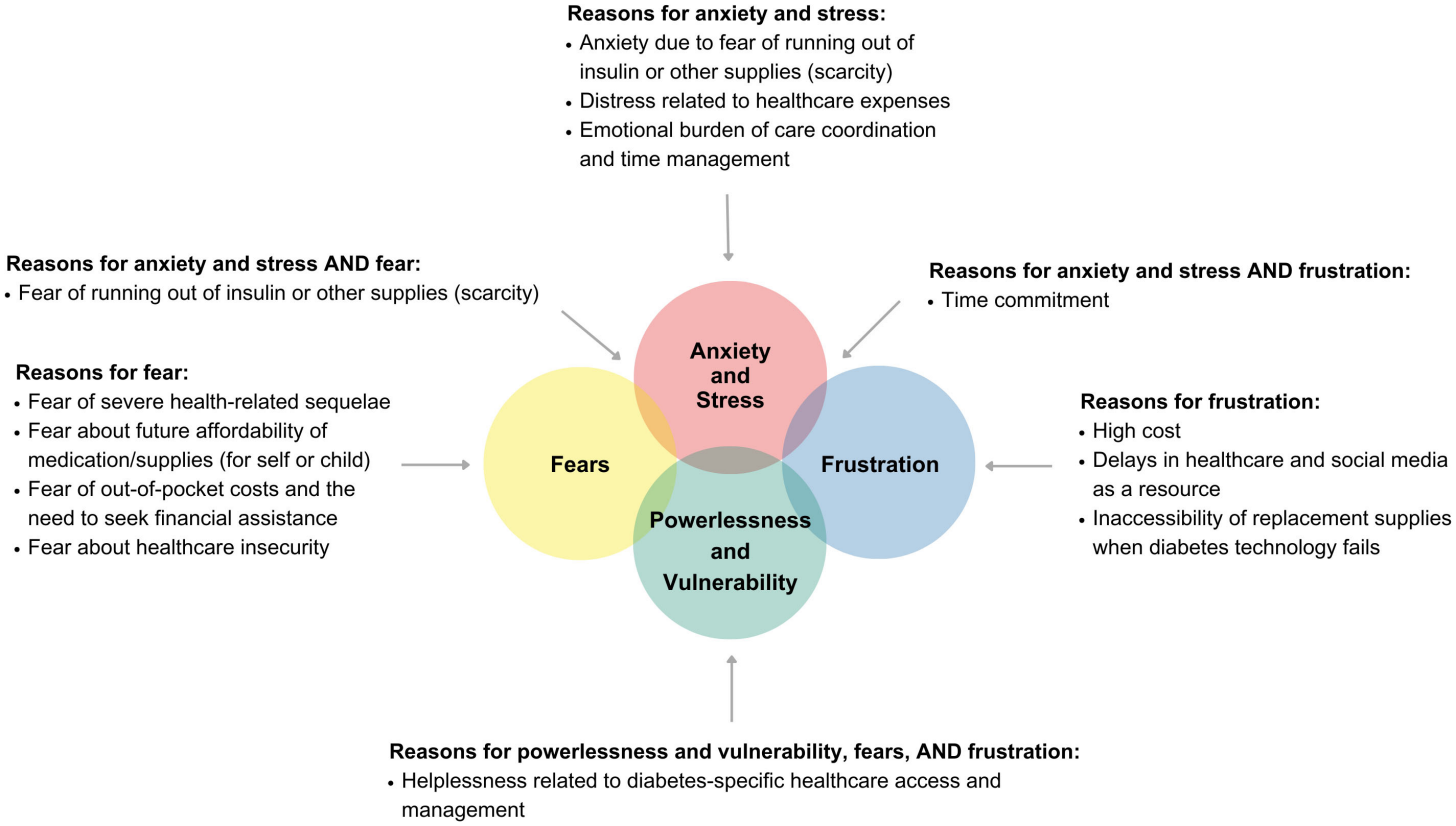
Perceived stress related to acquiring diabetes medications and supplies.

Participants reported significant stress related to navigating healthcare to access their DMS (**Figure 4**). Nearly half (47%) reported that their A1c levels were affected by their ability to acquire DMS. Just over half of participants (57%) felt they paid a fair price for insulin, and even fewer (37%) felt they paid a fair price for supplies, including diabetes technology. In this cohort, 37% of people requested financial help for diabetes costs from their family or legal guardians (**Supplementary Data Sheet 1**, **Table 2**).

### Theme 1: Anxiety and stress related to obtaining diabetes medications and supplies

Participants expressed immense stress and anxiety that stemmed from fear of running out of insulin or other supplies and the time commitment and financial burden of managing their condition. The struggle to afford insulin and other medically necessary supplies and the uncertainty of accessing healthcare resources created feelings of anxiety due to scarcity. Participants felt deep distress managing the day-to-day aspects of diabetes is challenging but controllable, while the bureaucratic and financial struggles were beyond their control. The fear of being unable to afford supplies, the need for constant vigilance in dealing with pharmacies and insurance, and the lack of control over essential aspects of their lives contributed to a pervasive sense of insecurity and anxiety among participants.

#### Subtheme: Anxiety due to fear of running out of insulin or other supplies (scarcity)

Anxiety due to scarcity emerged as a large stressor for participants. When participants were running low on insulin and/or other supplies they felt panicked, overwhelmed, and hopeless and began looking for immediate solutions including rationing and other related behaviors. When asked about what they were feeling at a time that they came close to running out of insulin, one participant described the following:

“I think I just panic. I think also just … going through that … laundry list of … actions that I needed to take …’Okay, what comes first? …you’re going to have to eat less. Okay, what comes next? Okay, you’re going to have to, you know, do this and this.’ Or like, ‘Okay, … maybe go to the gym twice a day now so … you’ll be more insulin-sensitive,’ … all this … weird like doomsday prepping.” (29-year-old female).

#### Subtheme: Distress from healthcare expenses

Participants described distress arising from the high cost of diabetes medications, highlighting how expenses cause financial strain and force them to sacrifice joyful activities to afford medications.

“It’s stressful and it’s heartbreaking. It’s stressful realizing that your day-to-day is based on dollar signs, and more so than I’d say the general population, it’s not just about … how nice of an apartment can I afford; it’s can I afford to live today.” (35-year-old female).

“It’s also heartbreaking to see the way that people don’t understand what else is going on, on the underside, because if you look at me, I’m a healthy adult living my life. And so … when people are aware, to some extent … what is our household income, what is our family dynamic and they say well … why don’t you just do X, Y and Z and … well, I can do these fun things, or I can buy insulin.” (35-year-old female).

#### Subtheme: Emotional burden of care coordination and time management

Participants described feeling overwhelmed by the significant time commitment required to coordinate care and supplies, extensive time spent waiting on hold, making multiple phone calls, or be transferred to different people, all while juggling other responsibilities.

“And the first one is stress, just incredibly stressful … And it’s a commitment … I need to be able to block out time … at least half hour 45 minutes in my day track down all the proper pieces and the people that I need to be in contact with to make sure that everything is set up the way it needs to be” (32-year-old female).

Others shared feelings of stress about the additional time and effort required to access diabetes management tools.

“I hate having to make phone calls like this that are determining if I stay alive…. Just the anxiety alone of having the conversation is something that I’ve personally had to work through … in therapy in my life…. how to manage stress and anxiety around … practicality and logistics of diabetes … has been a big part of my life” (21-year-old female)

### Theme 2: Fear of health problems and financial consequences

Participants reported experiencing fear about becoming severely ill to the point of hospitalization, sustaining avoidable harm to their health, experiencing DKA, or facing potential death by rationing because of limited access to insulin and/or other supplies. Additionally, participants reported a fear of not being able to afford insulin and/or other diabetes supplies in the future, even if their finances were stable at the time of the interview. Many described frustration and fear that their medication coverage was dependent on the insurance provided by their employer. Participants also described fear of running out of supplies, fear of having to pay a steep out-of-pocket cost if their necessary supplies were not covered by insurance or if they unexpectedly lost their insurance, and fear of paying an exorbitant hospital bill if they had exhausted all other options to obtain insulin and were forced to go to the emergency room. Participants who are parents of diabetic children reported worry and fear of handing off the costs and management of obtaining medication and supplies to their children when they are of age.

#### Subtheme: Fear of severe health-related sequelae

Participants expressed overwhelming fear when they came close to running out of insulin or other supplies. Participants described death as a realistic potential consequence, and related fears of emergency healthcare costs and financial strain on their family.

“I was afraid that my daughter would die, because … she didn’t have the things that she needed … I even looked into how much insulin cost to make and how much insurance companies or medical companies … are charging for the insulin, and it made me sick to see what the differences in between the cost to make it, and how much it’s being sold for and … to know that they had my daughter’s life literally … in their hands.” (Parent of 6-year-old girl)

“The fears?… what if I run out of insulin? What if I get really sick? What if I go into DKA, and then I end up with like a $50,000 hospital bill, and I can’t pay that, and I’m just in, you know, in debt for the rest of my life?… I mean, of course, there’s always like the fear of … getting sick and dying” (35-year-old female)

“I fear that … I might … die because I don’t have … insulin with me or like I might go to the hospital, and … if it wasn’t for having health insurance,… I know that would put my family at like a financial strain … Like what if I do die because I don’t have the things that I need, and no one wants to help me?” (23-year-old female)

Participants also described fear of potential consequences of rationing, specifically hospitalization.

“I definitely had the fears that … I’m going to end up hospitalized, I’m going to end up hurt … because … when I’m splitting doses and purposely … trying to make strips last longer … it’s definitely taking risks with health and safety.” (25-year-old female)

#### Subtheme: Fear about future affordability of medication/supplies (for self or child)

Participants described ongoing fear about future affordability of medication and supplies. Many shared that, despite having stable finances, they feared an unforeseen healthcare issue or other financial emergency could disrupt their ability to afford diabetes medication and supplies. Further, caregivers of PWD expressed fear of their children would not being able to afford medication and supplies when they became too old for their parents’ health insurance coverage.

“I’m always afraid … that something will happen … with my job or my insurance or … going to the hospital unexpectedly, and all of the sudden … my plan of paying $100 out of every check won’t be enough … I’ll end up needing more … having to hit up a family member for a loan or something just because I am out of money.” (31-year-old female)

“There’s a lot of fear that comes with not having access to your supplies … because, like I mentioned, you could do everything right. You could have a healthy A1c. You could take care of yourself. You could eat well. You could exercise and see your doctor regularly. (emotional) But for Americans … for the most part, as a diabetic … you’re at the mercy of your employer and the healthcare option they choose to you as to whether or not you have access to what you need, which feels very unfair.” (30-year-old female)

#### Subtheme: Fear of out-of-pocket costs and the need to seek financial assistance

Participants stated that they were afraid of having to pay unanticipated medical bills and shared fear of having to reach out to others for financial support.

“Fear, I would have to go to the hospital and then have another ginormous bill to pay,… or fear that I would have to … ask too big of a favor of somebody else to save my life and just being a burden … is a big part of that…. That is debilitating in a way that like … I would do everything I could before I asked somebody else to give something away…. I would go through … everything I could possibly do on my own before asking for help, and that is like the worst feeling to have.” (23-year-old female)

#### Subtheme: Fears about healthcare insecurity

The connection between medication affordability and life choices, including employment and relationships, emerged multiple times. Participants shared fears of losing or changing insurance due to losing their job or aging out of their parents’ insurance.

“I want to change jobs, but … what if … I get another job, and I find out that I’ve got another high deductible insurance, and I can’t get the medication I need. It feels like every decision that I make for myself in my life and my goals and my career … all comes down to how am I going to survive … it does feel like … I don’t have a solid ground beneath my feet.” (35-year-old female)

“I never have because I always feel like, from the times that I’ve been underinsured … there is always that feeling of like I need to hoard these supplies in case something happens. Or like what if I lose my job, and I don’t have enough supplies?… so, I do tend to use those a lot longer … than I should, but it does keep a little stock for me … those times that I kind of have to go longer without.” (35-year-old female)

### Theme 3: Frustration with the healthcare system

Frustration emerged as a recurring theme, stemming from challenges in accessing necessary supplies and navigating insurance issues. Participants expressed frustration with insurance coverage limitations, pharmacy processes, difficulties with suppliers, and the financial burden of managing diabetes. The complexity of obtaining essential supplies and the amount of time dedicated to obtaining supplies exacerbated feelings of frustration. Persistent uncertainty surrounding access to supplies created ongoing frustration.

#### Subtheme: Frustration due to the high cost of diabetes medications and supplies

Many participants expressed frustration with the high cost of insulin and other supplies. Participants lamented the sacrifices they were forced to make due to the financial burden of medical costs. Many described a sense of unfairness in having to pay exorbitant prices for life-sustaining medications, drawing comparisons to medications used in other conditions that cost less or to countries where insulin and diabetes supplies are either more accessible or free.

“[It’s] definitely frustrating to have to make sacrifices over something that’s gonna … keep me alive. But, unfortunately, as time passes, you just kind of accept your fate … I have no other choice.” 26-year-old female

“It’s frustrating that we have to pay so much when this isn’t her fault. It was just bad luck that she developed this…. I know she’s already started thinking about her future and being able to afford all of the supplies and worried about once she’s out on her own, if she’ll be able to afford to stay on a pump” (Parent of a 17-year-old child)

“[Getting supplies is] definitely the worst … anxiety I have to deal with because I think a lot of people would think changing your diet or taking shots would be the worst part of it, but that’s honestly something that becomes routine…. You do it every day, and it just becomes part of your life. (emotional) But you don’t ever really get over the frustration of having to pay $300 for a vial of [insulin] that you need to survive, and you don’t ever get over the frustration (crying) of having to be in medical debt because you want to live…. That pain and that frustration is something that I don’t think people understand.” (30-year-old female)

“The hardest frustration isn’t the diabetes itself. You can come to an acceptance of that. The hardest thing is living in a country where … you’re told that this is the best place to live, but other countries have access to these things for free that you have to spend thousands of dollars on or that you can’t get at all. That’s something that I think, for most adult diabetics is, by far,… the biggest … mental hurdle that you have to get through every day.” (30-year-old female)

#### Subtheme: Frustration with delays in healthcare and social media as a resource

Participants shared stories of frustration with spending long periods of time on the phone with insurance, pharmacies, and durable medical equipment suppliers. They highlighted the often unseen struggle of obtaining prior authorizations, emphasizing the repetitive and time consuming back-and-forth communication required between various healthcare entities to secure approval for DMS. To bypass these obstacles, many turned to social media platforms leveraging connections within the diabetes community to more quickly and efficiently obtain the supplies they needed.

“In any given month, I probably spend two hours on the phone with the healthcare provider or an insurance company, and I go to my endocrinologist once every three months. So the amount of time lost to being a diabetic that other people don’t have … It’s something that impacts you financially. It’s something that impacts your time and your health and every aspect of your life. So I think it’s an unseen thing, and that’s frustrating as well.” (30-year-old female)

“The biggest frustration is that it’s not always in a timely manner so often it’s ‘okay now you wait on hold’ or I leave a message and two or three days before they call back.” (32-year-old female)

“About a year ago … when I was at my job, my insurance plan was changing…. they were no longer going to offer the type of insurance that I was on. So, I had to switch to a new plan … and that new plan was the absolute worst. And it was one of those plans … [a Health Maintenance Organization], that … they needed [prior-authorizations] for basically every single thing…. I needed more … sensors, and they wouldn’t be able to give them to me until I had [prior authorizations], which took forever.… It was really frustrating, and I wasn’t able to get those things. So that was another time that I reached out to a [social media] group and asked if anybody had any spare sensors.” (25-year-old female)

#### Subtheme: Frustration with inaccessibility of replacement supplies when diabetes technology fails

Participants were frustrated by their inability to afford or obtain spare supplies in case of unexpected issues or emergencies, resulting in rationing of pump or CGM supplies

“And then for my insulin pump, I really started [rationing supplies] because … I was getting so frustrated…. It doesn’t happen regularly, but if [an insulin pump] site were to fail or if I … [detached it from my body] or something and I know I didn’t get the full three days out of it, I would … look at that as money just going in the trash can. Well, that’s a whole sensor, a whole cartridge that I have to throw away, and I didn’t even get 100% use from it.” (25-year-old female)

### Theme 4: Feelings of powerlessness and vulnerability

Participants frequently discussed feeling little to no control over their own care within the healthcare system. Participants explained that even if they followed all of the recommended care instructions for managing diabetes, ultimately, their health was limited by their level of access to insulin and other supplies, which is determined by insurance and other healthcare entities.

#### Subtheme: Helplessness related to diabetes-specific healthcare access and management

When participants were asked, “How does stress caused by getting your DMS compare to other diabetes stressors?”, most participants expressed that the stress of obtaining DMS was more than day-to-day management. Others described the two stressors as different types of stress. One participant described the stress of obtaining supplies as “existential” because she would need to navigate a healthcare system that is largely out of her control for the rest of her life.

“Whatever the diabetes problem is in that day-to-day is under my control. It’s something I can think through and solve by myself…. But medications and things through the government and things through insurance is completely out of my hands, and I don’t feel like I have a voice in that problem-solving system.” (23-year-old female)

Another reported not being able to obtain health insurance on her own due to having multiple disabilities, limiting their ability to work and acquire health insurance.

“I also just feel like the affordability of supplies shapes our whole lives…. I know I already have maxed out the point I have to be married by because I’m multiply disabled in many other ways and can’t really hold … many jobs, and the jobs I do hold aren’t the kind that offer health insurance. … Even if my partner and I ideally would have liked to wait a little longer [to get married], I know when I have to be married by so … I have health insurance when I turn 26.” (32-year-old female)

Overlapping themes of frustration and powerlessness emerged during the interviews. Participants reported frustration about the bureaucratic nature of the healthcare system and that patients and clinicians are at the mercy of profit-driven insurance companies.

“I think there’s too many layers of bureaucracy that’s gotten involved, and there’s too many hands … trying to make a profit…. Why do I have to use a third-party supplier? … That’s just [an] extra layers that don’t make sense…. Insurance is negotiating these contracts in the best interests of insurance, not in the best interests of the patient, and then insurance tells us who we’re allowed to use based on that contract. But if insurance can get a contracted price with a third-party supplier that’s better than what they can provide at [an insulin pump company], then somebody needs to be looking at what the [profanity] is happening at [the insulin pump company] … At the end of the day, the doctors lost the control and the power to decide what the patient needs, and the patient has lost the control and the power to advocate for themselves and get what they need.” (Parent of a 12-year-old child)

The pervasive sense of powerlessness among participants highlights the profound impact of systemic barriers on their ability to manage diabetes effectively.

### Limitations

Our study is limited by the limited diversity of our participant sample, primarily non-Hispanic, who identify as female with access to technology, and lack of diversity of analysts for qualitative interviews. There are inherent limitations with qualitative research, including limited generalizability, subjectivity and bias, and participant bias. However, we used numerous methods to increase rigor, including professional transcription services, multiple coding with consensus review, reflexivity, and decision tracking.

## Discussion

Our findings underscore the significant emotional burden associated with securing necessary DMS among people with T1D and their caregivers who turn to social media as an alternative source of life-saving medication and equipment. The emotional experiences were marked by intense psychological distress, including a profound fear of running out of insulin, deep concerns over future affordability, and anxiety over the precariousness of access tied to employment. Participants voiced deep frustration with the healthcare system, feeling helplessness in navigating its complexity, and expressed fears of severe health consequences, including illness, hospitalization, or even death. These findings bring to light the scarcity and uncertainty faced by PWD, suggesting that similar challenges may apply to other groups.

While extensive research has emphasized the negative impact of diabetes distress (9–12), which comprises worries and fears about potential complications, unexpected fluctuations in glucose levels, and hypoglycemic episodes (13, 14), there is limited data on the stress of encountering barriers to medication access within the healthcare system (15, 16). Diabetes distress has been linked to lower engagement with diabetes self-management and higher hemoglobin A1c levels in T1D (17), while reductions in diabetes distress showed improvements in both domains (18). In our study, the majority of participants noted that managing DMS was *more* burdensome than managing T1D itself. This underscores the need for further research into the systemic challenges patients face and the unseen emotional toll of navigating healthcare barriers.

Strong emotions related to the high cost of prescriptions arose as an important theme throughout interviews. This finding was corroborated by a 2022 survey of 287 18–30-year-olds with T1D, where 89.5% of participants endorsed the statement “I worry about the cost of diabetes.” In the same study, participants described diabetes costs as “all-consuming” and a “source of fear,” similar to our findings (19). These high out of pocket costs have been reported in by Chua et al., who estimated the mean annual out of pocket costs were estimated to be $2,298 for children and $2,414 for adults with T1D (20). Additionally, a 2017 study from the American Diabetes Association found that PWD who are uninsured without consistent access to insulin end up with 168% more emergency department visits than people who have insurance (21). Thus, the fears expressed by our participants are valid and barriers to access may be associated with substantial health consequences. In recent years, several states have implemented legislation capping copayments for insulin. The federal government passed the Inflation Reduction act of 2022 to cap the out of pocket cost of insulin at $35 per month for Medicare beneficiaries, which led to more insulin prescriptions being filled (22). However, many people with diabetes who rely on insulin to survive are not covered by these initiatives.

On top of the cost, PWD often act as as a intermediaries between various healthcare entities, including health insurance companies, DME suppliers, pharmacies, and clinics (23). Our findings indicate that PWD experience frustration with the significant time commitment needed to communicate with these organizations, with many participants specifically citing prior authorizations as a major cause of delays in obtaining their DMS. To address these issues, a comprehensive approach must be adopted to not only validate patients’ experiences but also help PWD learn to manage barriers that arise and address the underlying systemic challenges. To help patients navigate financial and insurance challenges, Blanchette and colleagues collaborated with the T1D community to create a T1D Health Insurance and Financial Toolkit (24) to provide education for young adults about financial stress (26) and health insurance. Such resources are imperative to help PWD navigate insurance, learn actionable problem-solving skills, and improve self-management and psychosocial wellbeing. Additionally, in recent years the American Medical Association has been advocating for several state and national reforms to streamline prior authorization processes (25). Continued efforts are needed to alleviate the burden on patients by reducing delays in care and minimizing administrative complexities that impede timely access to treatment.

Moreover, it is crucial to have established procedures that allow PWD to openly address obstacles they encounter, as these conversations may be difficult for patients to initiate. Implementing universal screening that identify such barriers can streamline the process. Furthermore, involving community health workers, patient and insurance navigators, social workers, case managers, and pharmacists can provide comprehensive support for financial and practical patient concerns. For some patients, psychological support may be necessary, so making appropriate referrals to mental health specialists can help ease the emotional burden. In any case, creating a non-judgmental environment where patients feel heard and validated is essential.

While addressing the psychological stress experienced by patients is important, the root cause of these barriers is a diffusely flawed healthcare system that ultimately does not ensure PWD have access to the DMS they need to survive. The Theory of Access framework originally developed by Penchansky and Thomas (27) and modified by Saurman (28) and Litchman and colleagues includes seven dimensions of access: accessibility, availability, acceptability, affordability, adequacy, awareness, and association. If these dimensions were all met, there would be no need for support through social media. Some of these dimensions — accessibility (location), availability (supply and demand), affordability (financial and incidental costs), and adequacy (organization of current systems) — change frequently over the lifetime of a PWD (5). Thus, policymakers need to address the underlying issues in our healthcare system that are causing disparities in access.

## Conclusion

People affected by diabetes experience significant emotional and psychological distress due to barriers to accessing necessary insulin and diabetes supplies. The soaring costs of insulin, the complexities of navigating the healthcare system, and the deficiencies in current healthcare policies contribute to a pervasive sense of fear, anxiety, and frustration among PWD. The reliance on non-traditional methods, such as underground exchanges and social media, to obtain essential medications underscores the urgent need for systemic changes. It is evident that while educational resources can help PWD better navigate these challenges, more robust policy interventions are critical to mitigate the emotional burden on PWD and foster a more just healthcare environment.

## Data Availability

The raw data supporting the conclusions of this article will be made available by the authors, without undue reservation, upon request.
