# Supplementary material for: “Can I afford to live today?” The emotional toll of navigating the healthcare system with type 1 diabetes
Source: Front Endocrinol (Lausanne). 2025 Mar 24;16:1555265. doi: 10.3389/fendo.2025.1555265 (PMC11973976; doi:10.3389/fendo.2025.1555265)
Supplement: Supplementary file 1 [file DataSheet1.pdf]

Navigating the Healthcare System With Diabetes: Supplemental Data

1. Interview Guide

Purpose: Describe real-life experiences of people with diabetes (PWD) relating to access and affordability of diabetes medication and supplies.

Population: Individuals in the United States who have reached out to social media groups to find support for obtaining or affording diabetes medications and supplies.

Introduction: Thank you for agreeing to take part in this interview. My name is XXX. I am a researcher trying to better understand how access and affordability of medication and supplies affects people with diabetes. I want to hear about your real-life experiences about accessing and affording medications and supplies.

Your participation is extremely valuable and could help improve our understanding of what it's really like to manage diabetes. There are no right or wrong answers. You do not have to answer any question you don't feel comfortable answering.

I would like to record our conversation to make sure I transcribe what you actually say and not my own interpretation of what you say. Your responses will be written down word-for-word using the recordings. Your answers may be used in research reports, but your name or any identifying information will not be associated with your responses. Before we begin, do you have any questions for me?

Interview Questions (with probing bullet points):
*Bold = ask in every interview, non-bold = ask if more info is needed

Warm-Up Question:

Just to get me familiar with you and your experiences. . .

1. **Tell me a little bit about what supplies and/or technology you use to manage your diabetes?**
    a. Do you use injections, an insulin pump, an insulin pen, or a CGM?
    b. What types of insulin do you use?

Broad Questions:

Now, I'd like to ask a few specific questions about your diabetes medication and supplies.

1. **How did you find the social media group that you reached out to? Which one did you reach out to? Tell me the story of how you came to reach out to [social media group]? What led you to reach out? How did the social media help you (tell me more about the type of support – how did that go)? Have you ever reached out to manufacturers for assistance programs to help you get diabetes**

medications/supplies? How did that go? Have you ever traded medications/supplies with other people with diabetes? How did you go about that (how did you find others with supplies)?

   a. **Tell me about any issues you have had getting your diabetes medication and/or supplies? What medications have you had trouble getting?**
      i. Tell me a story about a time when getting your medications was challenging.
      ii. What steps did you take to try to get these diabetes medications?
      iii. What was helpful in this process? What made things harder for you? How could this process be made easier for you?
   b. **What supplies have you had trouble getting?**
      i. Tell me a story about a time when getting your meds/supplies was challenging.
      ii. What steps did you take to try to get these diabetes medications/supplies?
      iii. How could this process be made easier for you?
   c. **What do you think has caused these issues?**
      i. What makes the supplies hard to afford for you and your family?
      ii. What makes the supplies hard to get (other than cost) for you and your family?

2. **Walk me through how you order your medications and supplies. You may use your last order as an example.**
   a. **Can you tell me about a situation where you experienced delays in getting your medications/supplies?**
   b. [Ask if not mentioned] Do you typically have to contact your doctor's office more than once? Do you have to contact anyone else (pharmacy, DME supplier, etc.…)?
   c. **How long does it take to obtain your medications and supplies after you request them? Tell me more...**
   d. How do you pay for your diabetes medications/supplies (i.e. copays, patient assistance programs, coupons, donations from friends/family, GoFundMe, etc.).

3. **Have you ever tried to stretch, conserve, or extend use of your medications or supplies or make them last longer? That's not medically recommended**
   a. **What steps do you take to make them last longer? What made you decide to do this (Is there anything else that made you decide to do this)? How did that feel? How did it affect you?**
   b. **Do you do anything to avoid wasting your medications or supplies? What do you do? How does doing this make you feel?**
   c. **Have you had to make any changes to your life or tradeoffs to afford or obtain your diabetes medications/supplies?** Can you give me some examples? How did you decide to do that? How did that make you feel?

d. How have the challenges or costs related to getting diabetes medicine and supplies impacted other parts of your life? (i.e. changes in how you **spend money**, changes in how you take insulin, changes in eating, changes in exercise or physical activity, etc.). **How did it affect you and your family, your job, etc.?**

4. **Tell me about a time when you came close to running out of insulin or other supplies?**
   a. **What fears did you have?**
   b. How did you get through the situation?

5. **Have you gotten sick, gone to the ER, or been hospitalized because you did not have the diabetes medications and supplies that you needed? Tell me about it.**
   a. **Did you have to miss work? How did your job respond?**

6. **What would you do, today, if you were running extremely low on medication or supplies?**
   a. Who would you reach out to?
   b. **What feelings come to mind when you think about managing and getting your medication and supplies?**
   c. **How does [stress caused by getting your diabetes medications and supplies] compare to other diabetes stressors? How has it changed over time?**
   d. **How do you feel about discussing these issues with your diabetes healthcare provider?** Have you discussed it with him/her in the past? If so, how did that go? What was helpful about it and what was challenging?

7. **What was your most recent A1c? Has that been affected by your ability to obtain diabetes medications and supplies? How so?**

8. **What do you think needs to be done to help you have better access to or be able to afford your diabetes medication and supplies?**

2. Additional Questions

1. On a scale of 1 to 5 with 1 being “No stress” and 5 being “Severe stress,” how much overall stress does obtaining diabetes medications and supplies cause you?
2. Do you receive financial help for diabetes costs from your family or legal guardians?
3. Do you feel like you pay a fair price for insulin?
4. Do you feel like you pay a fair price for diabetes supplies?

Supplemental Table. Participants were asked about ho in the study and the number that responded. Data are reported as n (%).

Question	No. Respondents
N (%) individuals felt that their HbA1c level was negatively impacted by their ability to afford and obtain diabetes medications and supplies	14 (47%)
N (%) individuals felt they paid a fair price for insulin	17 (57%)
N (%) individuals felt they paid a fair price for diabetes supplies	11 (37%)
N (%) individuals reported requesting financial help for diabetes costs from family or legal guardians	11 (37%)
